# Molecular characterization of flavanone 3-hydroxylase gene and flavonoid accumulation in two chemotyped safflower lines in response to methyl jasmonate stimulation

**DOI:** 10.1186/s12870-016-0813-5

**Published:** 2016-06-10

**Authors:** YanHua Tu, Fei Liu, DanDan Guo, LiJiao Fan, ZhenXian Zhu, YingRu Xue, Yue Gao, MeiLi Guo

**Affiliations:** School of Pharmacy, Second Military Medical University, Shanghai, 200433 People’s Republic of China; School of Biological and Environmental Sciences, Nanjing Forestry University, Nanjing, 210095 People’s Republic of China

**Keywords:** Flavanone 3-hydroxylase gene, Functional characterization, Flavonoid accumulation, Methyl jasmonate, Safflower (*Carthamus tinctorius* L.)

## Abstract

**Background:**

Among secondary metabolites, flavonoids are particularly crucial for plant growth, development, and reproduction, as well as beneficial for maintenance of human health. As a flowering plant, safflower has synthesized a striking variety of flavonoids with various pharmacologic properties. However, far less research has been carried out on the genes involved in the biosynthetic pathways that generate these amazing flavonoids, especially characterized quinochalcones. In this study, we first cloned and investigated the participation of a presumed flavanone 3-hydroxylase gene (F3H) from safflower (*Ct*F3H) in a flavonoid biosynthetic pathway.

**Results:**

Bioinformation analysis showed that *Ct*F3H shared high conserved residues and confidence with F3H from other plants. Subcellular localization uncovered the nuclear and cytosol localization of *Ct*F3H in onion epidermal cells. The functional expressions of *Ct*F3H in *Escherichia coli* BL21(DE3)pLysS cells in the pMAL-C5x vector led to the production of dihydrokaempferol when naringenin was the substrate. Furthermore, the transcriptome expression of *Ct*F3H showed a diametrically opposed expression pattern in a quinochalcone-type safflower line (with orange-yellow flowers) and a flavonol-type safflower line (with white flowers) under external stimulation by methyl jasmonate (MeJA), which has been identified as an elicitor of flavonoid metabolites. Further metabolite analysis showed the increasing tendency of quinochalcones and flavonols, such as hydroxysafflor yellow A, kaempferol-3-O-β-D-glucoside, kaempferol-3-O-β-rutinoside, rutin, carthamin, and luteolin, in the quinochalcone-type safflower line. Also, the accumulation of kaempferol-3-O-β-rutinoside and kaempferol-3-O-β-D-glucoside in flavonols-typed safflower line showed enhanced accumulation pattern after MeJA treatment. However, other flavonols, such as kaempferol, dihydrokaempferol and quercetin-3-O-β-D-glucoside, in flavonols-typed safflower line presented down accumulation respond to MeJA stimulus.

**Conclusions:**

Our results showed that the high expression of *Ct*F3H in quinochalcone-type safflower line was associated with the accumulation of both quinochalcones and flavonols, whereas its low expression did not affect the increased accumulation of glycosylated derivatives (kaempferol-3-O-β-rutinoside and rutin) in flavonols-typed safflower line but affect the upstream precursors (D-phenylalanine, dihydrokaempferol, kaempferol), which partly revealed the function of *Ct*F3H in different phenotypes and chemotypes of safflower lines.

**Electronic supplementary material:**

The online version of this article (doi:10.1186/s12870-016-0813-5) contains supplementary material, which is available to authorized users.

## Background

Flavonoids with a variegated structure are extensively scattered across most plants in nature and play pivotal roles in plant acclimatization to varying environments. For example, they provide a strong defense against insect infestation [1, 2] herbivores [3], and pathogenic microorganisms [4] and also conduct signal molecules to the environment to promote interaction with symbiotic bacteria [5] and viruses [6]. Pigmentosusflavonoids act as visual and olfactory hits to attract pollinators and herbivores and protect against light intensity, temperature, and biotic and abiotic stresses [7]. Moreover, they provide abundant resources for agents that promote and maintain health [8].

Because of their significance in genetic investigations and biomedicine, flavonoid biosynthesis has attracted considerable scientific attention over the years [9, 10]. The pathway of flavonoid biosynthesis has been basically clarified through enzymologic studies in the plant model *Arabidopsis thaliana* [11]. However, the secondary metabolic pathways in plants are extremely complex. Although the metabolic pathways in the model plant are understood, these pathways have different regulation mechanisms in another species or in a specific variety, especially when the flavonoids differ from those in the model plant. Therefore, it is essential to identify species-specific genes in the flavonoid biosynthesis pathway.

Safflower (*Carthamus tinctorius* L.) is not only cultivated as an oilseed crop but also widely used as a traditional medicine in China. Modern pharmacologic experiments have shown that safflower, along with its active compounds, has wide-reaching biological activities, including dilating the coronary artery, improving myocardial ischemia, and modulating immune responses [12, 13]. Many researches on phytochemistry have shown that safflower synthesizes a striking variety of flavonoids, such as quinochalcones (hydrosafflower yellow A, carthamin, tinctorimine, and cartorimine) and flavonols (kaempferol and its glucosides, and quercetin and its glucosides), among others [13]. Simultaneously, investigations on the genome and transcriptome of safflower have also been carried out, annotating the unigenes involved in the biosynthesis of flavonoids and fatty acids [14]. Thus far, the genes encoding isochorismate synthase, cinnamate 4-hydroxylase, oleoylphosphatidylcholinedesaturase (FAD_2_), phenylalanine ammonia-lyase and chalcone synthase in safflower have been cloned and characterized [15–17]. Nonetheless, studies on genes encoding enzymes related to flavonoid biosynthesis and the identification of their functions are still lacking.

**Fig. 1:**
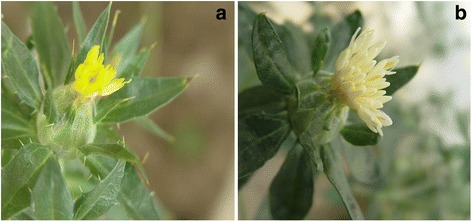
The two safflower lines: **a** ZHH0119 line. **b** XHH007 line

Flavanone 3-hydroxylase (F3H) is one of the nuclear enzymes acting at the bifurcation of the flavonoid biosynthetic pathway, initiating catalysis of the 3-hydroxylation of (2S)-flavanones, such as naringenin to dihydroflavonols [18]. The majority of F3H genes in the flavonoid metabolic pathway have been obtained from many different plants, including *Ginkgo biloba* [19], *Arabidopsis thaliana* [20], *Reaumuria soongorica* [21], *Reaumur trigyna* [22], *Triticum aestivum* L. [23], and *Camellia sinensis* [24], and subsequently characterized. However, no investigations on bioinformation analysis and functional identification of F3H gene in safflower have been reported. Meanwhile, no investigation on the cellular level of F3H enzyme has been done. To understand the flavonoids biosynthesis in safflower, two different safflower lines, a quinochalcone-type line with orange-yellow flowers and a flavonol-type line with white flowers were selected and conducted to research the F3H function and characterization in safflower (Fig. 1). The full length of CtF3H was cloned, and its open reading frame was inserted into the prokaryotic expression vector pMAL-c5X for protein expression and in vitro enzyme activity identification. Also, the recombination of *Ct*F3H and pCAMBIA-1380-CaMV35S-MCS-EGFP-NOS (PMT-39) was constructed to study its subcellular localization. Simultaneously, we investigated its transcriptome expression patterns and the accumulation of selected flavonoids in the corolla of the two chemotyped safflower lines (a quinochalcone-type safflower line with orange-yellow flowers and a flavonol-type safflower line with white flowers) under MeJA induction.

## Results

### Characterization of *Ct*F3H

**Fig. 2:**
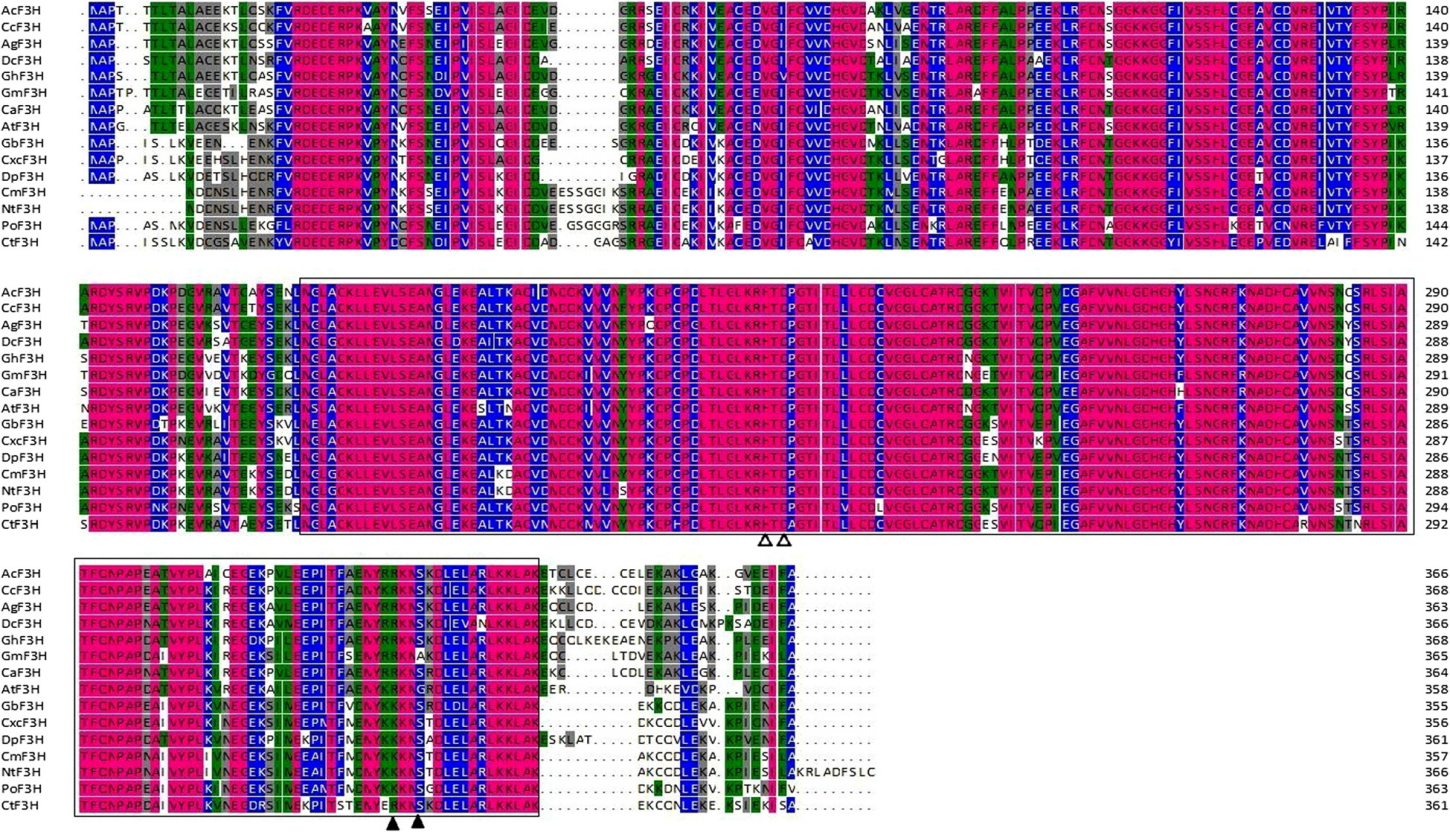
Alignment of CtF3H with F3H proteins from other species. Genbank accession numbers for the proteins in the alignment are as follow: AcF3H (ACL54955.1), CcF3H (Q05963.1), AgF3H (AFN70721.1), DcF3H(AAD56577.1), AtF3H (NP_190692.1), GmF3H (ACM62745.1), GhF3H (ABA01482.1), CaF3H (AEO36935.1), CtF3H (AEG64806.1), DpF3H (BA J21534.1), GbF3H (BAJ17667.1), CcF3H (AFC37250.1), PoF3H (ACB56921.1), CmF3H (AAB97310.1) and NtF3H (AAC15414.1). The sequences were aligned using DNAMAN software. The Fe^2+^-binding sites and oxoglutarate-binding sites are indicated by empty (∆) or filled (▲) triangles, and the conserved 2-oxoglutarate-Fe(II) oxygenase domain is shown in the black box

The cDNA of *Ct*F3H contained an 1086bp open reading frame encoding 361aa. The molecular weight and theoretical isoelectric points were 40.72 kDa and 5.57, respectively. The alignment of the predicted amino acid sequences indicated that the *Ct*F3H protein contained 2-ODD (2–oxoglutarate-dependent dioxygenase) superfamily conserved domains as other F3H proteins: the 2-oxoglutarate binding domain RxS (Arg289 and Ser291) and the ferrous iron binding site HxDxnH (His78, His121, His211, Asp213, His221, Asp 223, His265, His267, and His279) (Fig. 2). The conservation observed in these amino acids revealed that *Ct*F3H protein has a potential biological function. A phylogenic tree analysis indicated that *Ct*F3H is more closely related to F3Hs *Dahlia pinnate*, *Gynura bicolor* and *Pilosella officinarum* (Additional file 1: Figure S1).

### Subcellular localization of CtF3H

**Fig. 3:**
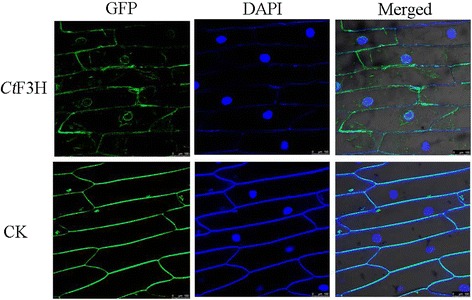
Subcellular localization of the GFP-*Ct*F3H fusion protein in onion epidermal cells. The fluorescence emission signals were determined by a confocal laser scanning microscope

The probable subcellular localization of *Ct*F3H was computationally analyzed by using the WoLF PSORT program, and the results indicated that *Ct*F3H may be localized in the cytoplasm or nucleus. To identify the exact localization of *Ct*F3H, the fusion construct of *Ct*F3H and GFP (Green fluorescent protein), controlled by the CaMV 35S promoter, and the GFP vector alone were introduced into onion epidermal cells through mediation of the Agrobacterium strain GV3101. As shown in Fig. 3, the onion epidermal cells infected with Agrobacterium harboring the *Ct*F3H-GFP protein emitted GFP signals predominately in the nucleus, cytosol and cytomembrane. However, onion cells transformed with GFP vector alone exhibited GFP signals in the cytomembrane. The results implied that *Ct*F3H was a nuclear and cytosol localized protein, giving some clues for its function in the biological processes of plant.

### Purification and in vitro activity of *Ct*F3H

**Fig. 4:**
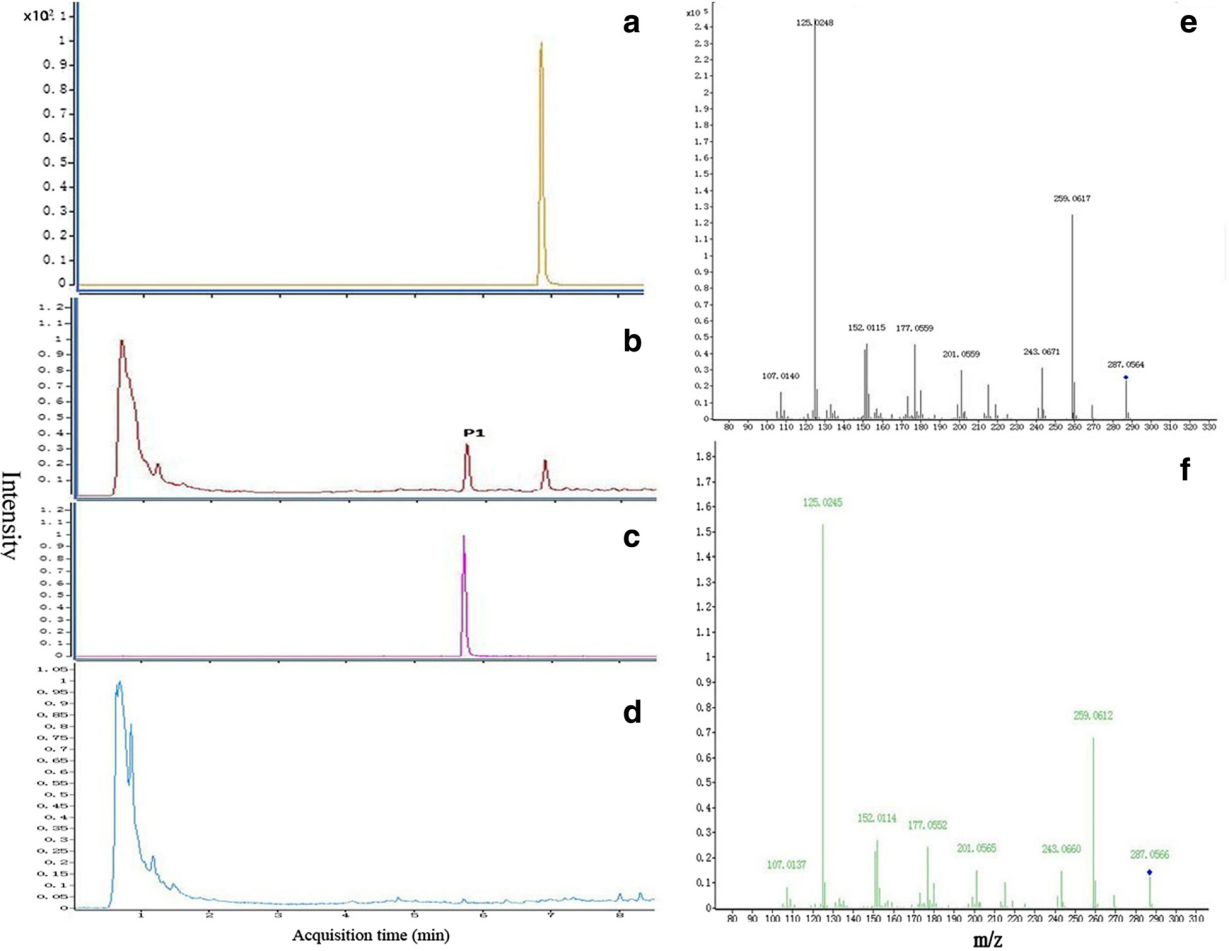
In vitro activity of recombinant *Ct*F3H protein without MBP-tag using naringenin as substrate. **a**-**d** UHPLC-MS-ESI profiles of naringenin, the reaction products catalyzed by *Ct*F3H protein using naringenin as substrate, dihydrokaempferol and solvents peak without *Ct*F3H protein and naringenin. **e**-**f** The mass spectrometry profile of reaction product (p1) and dihydrokaempferol standard

To show the enzyme activity of *Ct*F3H in vitro, crude recombinant protein expressed in *E. coli* was purified by using an amylase resin column. As shown in Additional file 2: Figure S2, lane 4, the *Ct*F3H protein without MBP (maltose binding protein) tag presented a molecular weight of about 40kDa, consistent with the size of the predicted *Ct*F3H protein. The in vitro activity of *Ct*F3H was also examined, with naringenin, kaempferol, dihydrokaempferol, quercetin and dihydroquercetin as the substrates. However, only naringenin could be catalyzed by CtF3H protein and generated other product. Other four compounds could not been catalyzed by CtF3H protein to yield other product. Our data suggested that one product (p1) was recognized apart from the naringenin and solvent peaks (Fig. 4a, b, and d), which shared the same retention time as dihydrokaempferol (Fig. 4c). The LC-MS/MS analysis indicated that the product had a molecular ion [M-H]^-^ at a mass-to-charge ratio (m/z) of 287, along with daughter ions with m/z 259, 243, 201, 177, 152, 125, and 107 (Fig. 4e); this is consistent with the product from the authentic standard of dihydrokaempferol (Fig. 4f). The result was also coincident with that F3H could convert naringenin to dihydrokaempferol from *Arabidopsis thaliana* [20].

The kinetic parameters of *Ct*F3H were tested by using a range of naringenin concentrations. The initial velocity V0 plotted against the naringenin concentration generated a rectangular hyperbola with V_max_ = (22.90 ± 0.94) μM/min and K_m_ = (43.75 ± 7.12) Mm (Additional file 3: Figure S3). Our data suggested that *Ct*F3H was effective in catalyzing naringenin into dihydrokaempferol, demonstrating its role in the pathway of flavonoid biosynthesis.

### Expression level analysis of *Ct*F3H in the flower tissue of two safflower lines

**Fig. 5:**
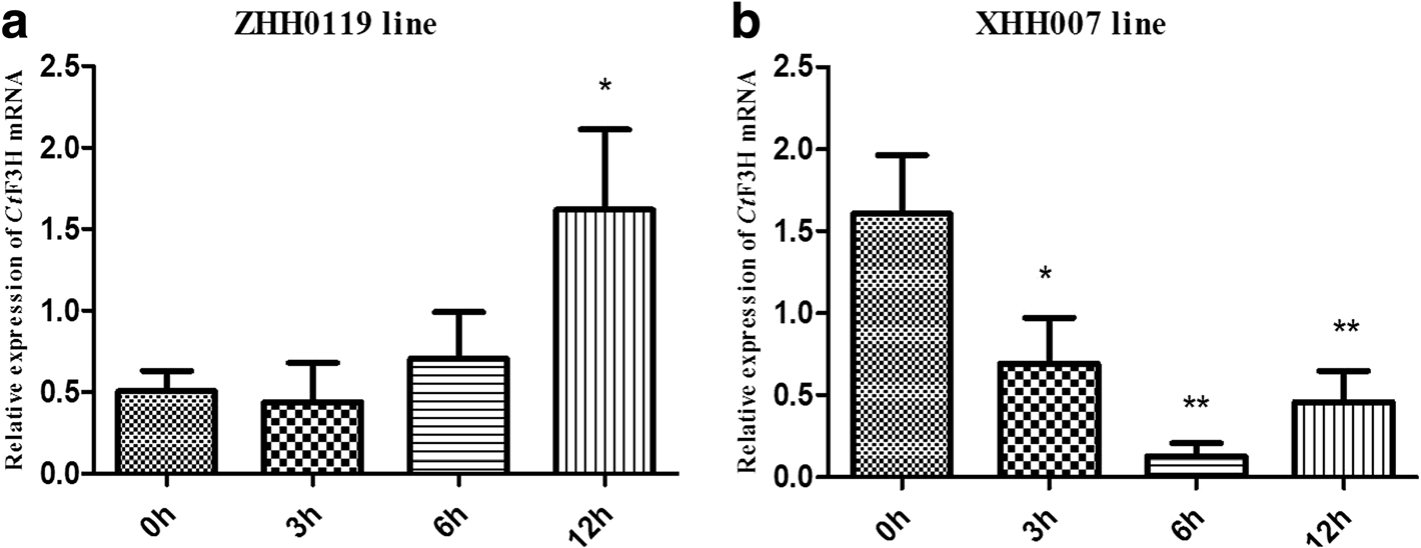
Quantitative real-time PCR analysis of *Ct*F3H relative expression of flower tissue in ZHH0119 line (**a**) and XHH007 line (**b**) at different time points post-treatment with MeJA. Each plant was individually assayed in triplicate (*n* = 4). Relative expression of *Ct*F3H gene is normalized to *Ct*60s as control and compared with the relative expression without MeJA treatment at different time points using 2-^△△Ct^ method. Asterisks indicate a significant difference from 0 h (*:*p* < 0.05, **:*p* < 0.01)

To emphasize the role of the *Ct*F3H gene in the pathway of flavonols in safflower and abiotic stress, *Ct*F3H was first selected to analyze its MeJA-stimulated responses in two chemotyped safflower lines (ZHH0119 and XHH007) by carrying out quantitative real-time PCR. As shown in Fig. 5a, the *Ct*F3H gene expression level in flowers of the ZHH0119 line gradually increased over time. Its expression level at 12 h after treatment was also distinctly higher than that at 0 h, 3 h, and 6 h after treatment, indicating statistically significant difference (*p* < 0.05). This result suggested that *Ct*F3H may play a role in the defense response of safflower. However, no significant effect of elicitor addition was observed at 3 h and 6 h compared with that at 0 h (*p* > 0.05). Contrary to its expression level in flowers of the ZHH0119 line, the *Ct*F3H gene transcript abundance in the XHH007 line was continuously suppressed by MeJA treatment over 12 h, indicating a statistically significant differences at 3 h, 6 h, and 12 h compared with that at 0 h (Fig. 5b). This result implied that the *Ct*F3H expression level in flowers of the ZHH0119 line was positively regulated by MeJA treatment, which in XHH007 line could be perturbated.

### Accumulation profiling of flavonoids in flower tissue under MeJA stimulation

**Fig. 6:**
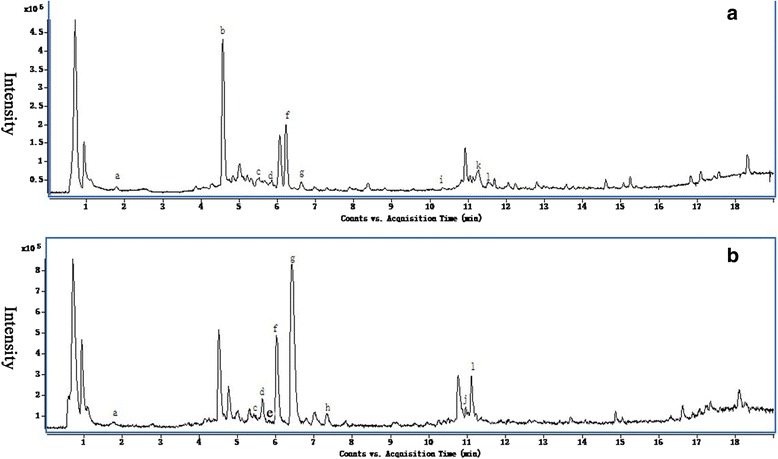
The total ECI profiles of ZHH0119 line (**a**) and XHH007 line (**b**). Detected compounds are labeled with letters. a, D-phenylalanine; b, hydroxysafflor yellow A; c, rutin; d, quercetin3-β-D-glucoside; e, scutellarin; f, kaempferol-3-O-β-rutinoside; g, kaempferol-3-O-β-D-glucoside; h, dihydrokaempferol; i, luteolin; j, apigenin; k, Carthamin; l, kaempferol

**Fig. 7:**
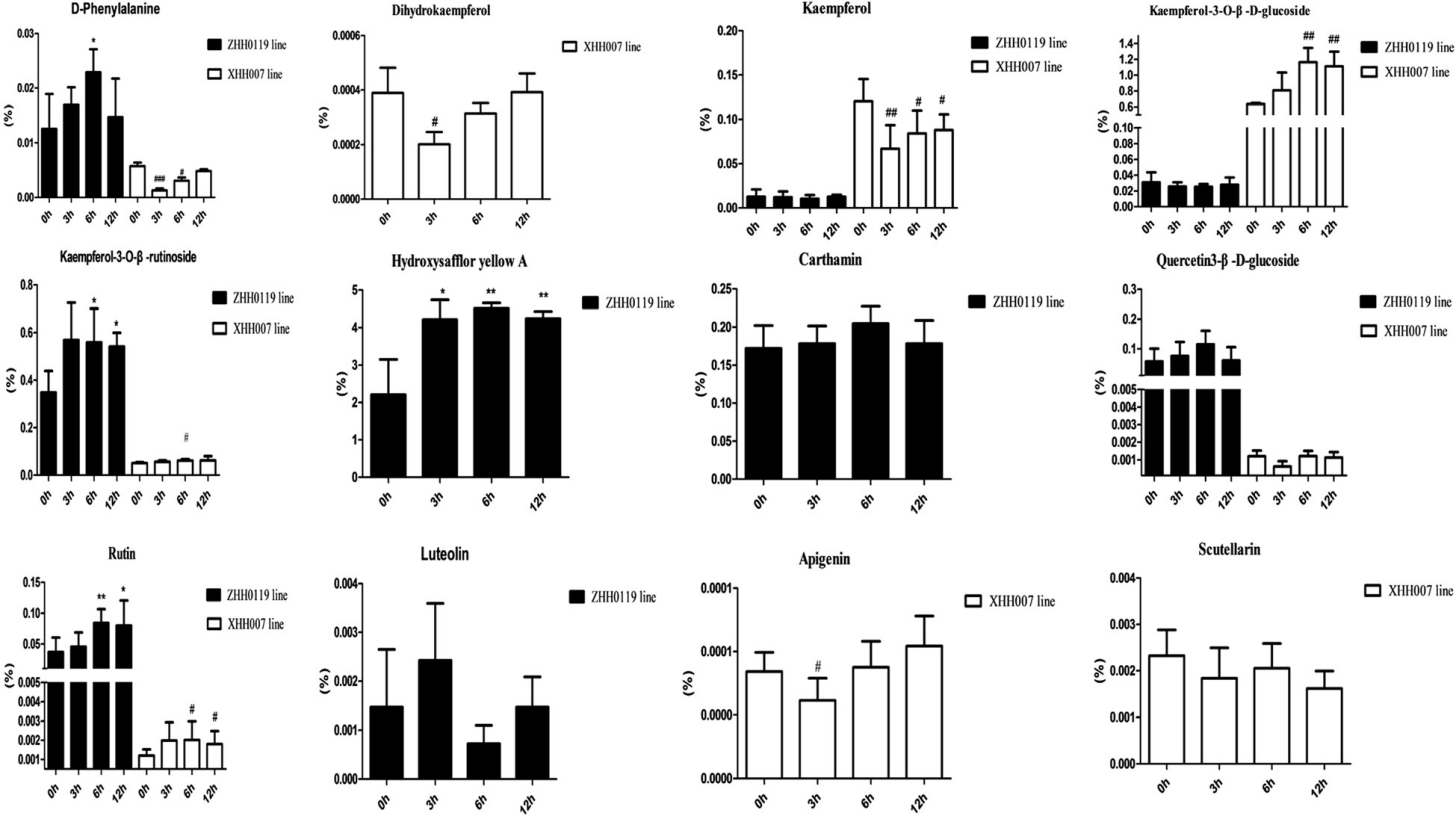
MeJA-induced changes of the targeted flavonoids accumulation in flower tissue of ZHH0119 line and XHH007 line. Asterisk indicates that the difference in ZHH0119 line is significant (*:*p* < 0.05) compared with that at 0 h. Hash mark indicates that the difference in XHH007 line is significant (^#^:*p* < 0.05, ^##^:*p* < 0.01) compared with that at 0 h

The extracted fragment mass ions of the authentic standards were as follows: hydroxysafflor yellow A: m/z 613.177 ([M + H]^+^), rutin: m/z 611.1627 ([M + H]^+^), quercetin3-β-D-glucoside: m/z 465.1038 ([M + H]^+^), scutellarin: m/z 463.0882 ([M + H]^+^), kaempferol-3-O-β-rutinoside: m/z 595.1675 ([M + H]^+^), kaempferol-3-O-β-D-glucoside: m/z 449.109 ([M + H]^+^), dihydrokaempferol: m/z 289.072 ([M + H]^+^), luteolin: m/z 287.0555 ([M + H]^+^), apigenin: m/z 271.061([M + H]^+^), carthamin: m/z 909.2104 ([M-H]^-^), and kaempferol: m/z 287.056 ([M + H]^+^). Profiles of the EIC (extracted ion chromatogram) are presented in Additional file 4: Figure S4. Processed by using the MassHunter qualitative analysis software (Agilent) for peak fitting, 9 targeted compounds were detected in flowers of the ZHH0119 line: hydroxysafflor yellow A, rutin, quercetin3-β-D-glucoside, kaempferol-3-O-β-rutinoside, kaempferol-3-O-β-D-glucoside, luteolin, carthamin, and kaempferol (Fig. 6a). Further comparative analysis revealed that the contents of these 9 targeted compounds in flowers of the ZHH0119 line showed different accumulation pattern under MeJA induction compared with that at 0 h (Fig. 7). Hydroxysafflor yellow A was a characteristic and dominant content in the ZHH0119 line, and its accumulation at 3h, 6h, and 12h consistently increased in response to MeJA stimulation, showing a statistically significant difference when compared with that at 0 h (*p* < 0.05). Carthamin, another quinochalcone-type compound in the ZHH0119 line, was positively regulated by MeJA stimulation. On the other hand, the flavonol-type content in the ZHH0119 line responsed to MeJA treatment also displayed dissimilar increased tendency. The accumulation of kaempferol-3-O-β-rutinoside and rutin were enhanced significantly both at 6 h and 12 h after MeJA treatment (*p* < 0.05). Nonetheless, accumulation of kaempferol-3-O-β-D-glucoside, kaempferol, luteolin and quercetin-3-β-D-glucoside displayed no statistically significant alteration responsed to MeJA stimulation. Regarding the accumulation in flowers of the XHH007 line, 9 identified compounds showed different accumulation tendency (Fig. 6b). Accumulation of apigenin and dihydrokaempferol were apparently depressed after 3 hour of MeJA stimulation, indicating statistical difference when compared with those at 0 h (*p* < 0.05). However, their accumulation at 6 h and 12 h were increased to the level that was no statistically difference with those at 0 h. Marked reduction were also observed on accumulation of kaempferol and D-phenylalanine. On contrast, accumulation of kaempferol-3-O-β-rutinoside and rutin were shown persistently up regulated pattern. In addition, accumulation of quercetin3-β-D-glucoside and scutellarin were discovered no statistically significant change, although quercetin3-β-D-glucoside at 3h after MeJA treatment represented decreased accumulation (Fig. 7). Kaempferol-3-O-β-rutinoside, kaempferol, rutin, quercetin3-β-D-glucoside and D-phenylalanine were recognized in both ZHH0119 and XHH007 lines. Except enhanced accumulation of kaempferol-3-O-β-rutinoside and rutin in both two safflower lines, kaempferol, quercetin3-β-D-glucoside and D-phenylalanine diaplayed converse or dissimilar accumulated pattern. On the whole, the relative content of kaempferol-3-O-β-D-glucoside, rutin and quercetin3-β-D-glucoside were lower than those in XHH007 line, which showed a converse result for the accumulation of kaempferol. These results suggested the accumulation patterns of flavonoids in two safflower lines respond to MeJA treatment were different, further indicating the potential formation mechanism of two phenotyped and chemotyped safflower lines.

## Discussion

Flavonoids, which accumulate abundantly in safflower, have been shown to have distinct biological properties. Various flavonoids have been separated and identified from safflower; however, their metabolic pathways still remain largely inexplicit. In the present study, we carried out a sequence analysis of *Ct*F3H and investigated its activity in vitro, as well as its transcript abundance and metabolic accumulation in two safflower lines treated with MeJA.

F3H is classified under Fe^II^ and 2-ODD [25]. All the F3Hs were featured with two conserved motifs: HxDxnH (His233, Asp235, and His289) for binding Fe^II^ and RxS (Arg299 and Ser301) for binding 2-OG (2–oxoglutarate) [26, 27]. These conserved residues were also disclosed in *Ct*F3H. Our research results also suggested that *Ct*F3H has a highly homologous relationship with F3H from other plants. These bioinformation analysis were suggested the potential function of *Ct*F3H in flavonoid biosynthesis pathway of safflower.

At the cellular level, flavonoids are deposited in various cellular compartments, such as the vacuole, cell wall, vesicles, cytosol, chloroplast, nucleus, and endoplasmic reticulum, and then integrated into proteins and nucleic acids to participate in crucial biological processes [28]. Flavonoid enzymes located in plant nuclei are believed to act as DNA protectors against UV and oxidative damage or to directly or indirectly regulate the transcription of genes that are requisite to growth and development [29]. In the present study, the results of subcellular localization implied that *Ct*F3H protein was localized to the nucleus and cytosol, which not only enriched but further supported the previous viewpoint [30], who pointed out that chalcone synthase and chalcone isomerase which are involved in flavonoid metabolism in Arabidopsis, are present in the cytoplasm and nuclei of some cells.

The analysis of its in vitro enzyme activity indicated that *Ct*F3H was effective in catalyzing the formation of dihydrokaempferol when naringenin was the substrate, which is compatible with the involvement of F3H in the flavonol biosynthesis pathway. The K_m_ and V_max_ at 30 °C were 43.75 ± 7.12 μM and 22.90 ± 0.94 μM/min, respectively. However, the K_m_ of *Ct*F3H (24 ± 3 μM) was higher than that of *At*F3H. These differences may reflect, to some extent, the unalloyed distinction of F3H genes between safflower and *Arabidopsis thaliana*, which may be attributed to their far evolution relationship from each other or to different reaction conditions, such as the temperature and reaction systems.

In nature, flowering is a crucial developmental process toward ensuring the survival and reproductive success of plants [31]. This process is affected by environmental and internal factors. MeJA, as a plant endogenous signaling molecule, has been shown to regulate vernalization, seed germination, root growth, flowering, fruit ripening, and senescence [32–35]. Also, MeJA has been identified as a predominant inducer of global genes involved in secondary metabolite pathways the accumulation of such genes in various plants, such as sweet basil [36] and *Medicago truncatula* [37]. As a flowering plant, the effect of MeJA on the transcript level of *Ct*F3H and the flavonoid production in flowers of two chemotyped safflower lines were uncovered. Plant phenotype is the ultimate result of gene expression difference regulated by strict spatial and temporal conditions [38, 39]. F3H gene mutations could affect color pigmentation, as previously noted in petunia, soybean, Japanese morning glory, carnation, and torenia [40–42]. In the present study, the transcript level of *Ct*F3H in a safflower line with orange-yellow flowers was gradually upregulated within 12 hours under MeJA application. In contrast, a gradual down regulation in a white-flowered safflower line was observed, suggesting a likely cause of colorless flower phenotypes. The converse expression pattern of *Ct*F3H in response to MeJA in two safflower lines may, to some extent, reflect the molecular mechanism underlying the different chemotypes of safflower. Moreover, many phenotypic characters of a plant depend largely on the accumulation of various metabolites in particular organs and tissues or at particular times in the plant life cycle following specific (internal and external) signals. A further analysis of the metabolite accumulation in the orange-yellow–flowered safflower line showed increasing contents of hydroxysafflor yellow A, carthamin, kaempferol-3-O-β-rutinoside and rutin. Kaempferol-3-O-β-rutinoside and rutin in white-flowered safflower line were also displayed increasing accumulation under MeJA induction. Except enhanced accumulation of kaempferol-3-O-β-rutinoside and rutin in both two safflower lines, other compounds in two safflower lines were shown converse or dissimilar accumulated pattern. Additionally, not all the selected compounds were recognized in both orange-yellow–flowered safflower line and white-flowered safflower line. These differential accumulation patterns of flavonoids may further indicate the formation mechanism in two phenotypes and chemotypes of safflower.

## Conclusions

To studies on genes encoding enzymes related to flavonoid biosynthesis and the identification of their functions in safflower, this study first cloned the F3H gene from safflower and then elucidated its function by in vitro enzymatic experimentation and analysis of its expression, as well as of flavonoid accumulation in response to MeJA treatment. The analysis of its in vitro enzyme activity indicated that *Ct*F3H was effective in catalyzing the formation of dihydrokaempferol when naringenin was the substrate, which is compatible with the involvement of F3H in the flavonol biosynthesis pathway. The results of subcellular localization implied that *Ct*F3H protein was localized to the nucleus and cytosol. The results of its expression and flavonoid accumulation in response to MeJA treatment showed that *Ct*F3H had converse expression patterns in two safflower lines under MeJA induction. Its high expression in orange-yellow–flowered safflower was related with the accumulation of both quinochalcones and flavonols, whereas its low expression did not affect the accumulation of flavonol in the white-flowered safflower, which partly revealed the function of *Ct*F3H in different phenotypes and chemotypes of safflower lines. These findings also implied that other F3H genes from safflower or transposable elements may positively regulate the accumulation of flavonols in white-flowered safflower. Our study thus provides a hypothetical and practical groundwork for increasing the flavonoid accumulation in safflower. Further investigations are needed to verify the function and roles of other *Ct*F3Hs in other safflower lines.

## Methods

### Discovery of CtF3H from the transcription profilingdatabase

To research the functional genes in safflower, the flower transcriptome sequencing was performed. The expressed sequence tags(ESTs) were annotated by BLASTx and BLASTn in the Nr and Nt database (unpublished). Only one gene was annotated as F3H in safflower flower.

### Plant materials

ZHH0119 line was collected from Chinese Safflower Germplasm Resources in Academy of Agricultural Sciences of Xinjiang. XHH007 line was from a cultivated variety bred by our laboratory and Academy of Xinjiang Agricultural Sciences and approved by the non major crop varieties registered office of Xinjiang in 2007. They were identified as *Carthamus tinctorius* L. by professor Meili Guo. The Voucher specimens of lines ZHH0119 and XHH007 were SMMU120625 and SMMU120626, respectively. They have been deposited in Medicinal Plant Herbarium of Department of Pharmacognosy, School of Pharmacy, Second Military Medical University.

ZHH0119 and XHH007 lines were grown in the greenhouse of the Second Military Medical University (Shanghai City, China). Both the ZHH0119 and the XHH007 lines were repeatedly purified in our laboratory. The ZHH0119 line, which has orange-yellow petals, is a major source of quinochalcones, whereas the XHH007 line, which has white petals, mainly contains flavonols without quinochalcones (Fig. 1). The plants were maintained at a mean temperature of 25 °C over a photoperiod of 16h.

### Isolation and cloning of *Ct*F3H

To obtain the full-length cDNA sequence of *Ct*F3H from safflower, 5′- and 3′-RACE experiments were carried out by using the SMART RACE cDNA amplification kit (Clontech, USA). The gene-specific primers TGATCCAACTCTCCCCACCATCACG (GSP-1) and GAGGGAGAACCGGTGGAAGATTGGAGG (GSP-2) were designed based on information on the *Ct*F3H fragment from the ESTdatabases of safflower in our laboratory. Amplified fragments of 5′- and 3′-RACE were cloned in the pMD-19 vector (Takara, Dalian, China) for sequencing. The primer pair *Ct*F3H-F/-R was subsequently designed based on the result of the sequence assembly (*Ct*F3H-F: CATCAACAAACACCCCACAC and *Ct*F3H-R: AGTGATAGCAACAAAAGCACAC) to amplify the full-length cDNA. The amplified fragment was cloned into the pMD-19 vector and then sequenced.

### Sequence analysis of *Ct*F3H

The sequence of *Ct*F3H mRNA has been submitted to GenBank under accession number AEG64806.1. The theoretical isoelectric points and mass values for the proteins were predicted by using the ExPASyProtParam tool (http://web.expasy.org/protparam/). To identify the conserved motifs of *Ct*F3H, its deduced amino acid sequences were aligned with F3H proteins from other species with the use of the DNAMAN software, version 8.0 [21]. The phylogenetic ralationships of F3H genes from dissimilar plants were shown through a phylogenetic tree constructed by using a neighbor-joining method implemented with the MEGA software, version 5.0. The parameters of the constructed trees were: phylogeny reconstruction: bootstrap method (1000 replicates), substitution model: amino acid and p-distance, substitutions to include: all, pattern among lineages: same (homogeneous), and rates among sites: uniform rates.

### Subcellular localization of *Ct*F3H protein

The subcellular localization of *Ct*F3H protein was first predicted by using the WoLF PSORT program (http://www.genscript.com/psort/wolf_psort.html) [43]. Then, the entire open reading frame of *Ct*F3H protein was amplified with primers by adding PMT-39vector-specific sequences on the 5′-ends(F:GAGCTTTCGCGGATCCGCCACCATGGCTCCGATATCGTCGT and R: CATGGTGGCAAGCTTAGGGCCGGGATTCTCCTCCACGTCACCGCATGTTAGAAG). The amplified fragment was then fused with the linearized PMT-39 vector by using the SunBio cloning Kit (Sun Bio, Shanghai, China). After being confirmed through sequencing, the generated recombinant t was transformed into the *A. tumefaciens*strain GV3101 by using an electroporation apparatus, as well as PMT-39 alone. Positive Agrobacterium were selected and cultivated in Luria-Bertani (LB) media supplemented with 50mg/L kanamycin and 100 mg/L streptomycin. After harvesting at an OD600 of 1.0, the Agrobacterium were centrifuged at 5500 rpm for 10min and resuspended in the same volume of MS liquid media. The onion epidermal layers were placed in the Murashige and Skoog(MS) liquid media for 20min and then incubated on MS solid agar with 0.4mol/L mannitol at 25 °C in the dark for 24 hours. Extraction of nuclei and 4’,6’-diamidino-2-phenylindole (DAPI) staining were carried out [44]. The GFP fluorescence of *Ct*F3H protein and the control were observed under a confocal microscope (Leica TCS SP5).

### Expression of recombinant *Ct*F3H protein, purification, and activity assay

The coding region of *Ct*F3H was amplified from the pMD19 (Takara) into the pMAL-C5x vector (NEB, New England) by using KOD DNA polymerase (Toyobo, Japan) and the primer pair *Ct*F3H-F/R (forward: 5′-CAAAGAACGTGCcatatgAAACCTAT-3′ with an added NdeI restriction site; reverse: 5′-TCTTAAGCAGATATTTTCTCGATGGGT-3′with an added EcoRI restriction site), which were designed from the sequence of *Ct*F3H mRNA submitted by our laboratory (GenBank accession no. AEG64806.1). The amplified product was sequenced and then digested with restriction enzymes (NEB, New England) to facilitate ligation with the appropriate cloning site of the linearized plasmid pMAL-C5x encoding a maltose-binding protein (MBP). After validation of its integrity, the construct was introduced into *Escherichia coli* BL21(DE3)pLysS cells (TransGen Biotech, Beijing) for protein expression.

Recombinant *Ct*F3H protein was expressed as described by the previous methods [45], with some modifications. *E. coli* BL21(DE3)pLysS cells containing the pMAL-C5x-*Ct*F3H were cultured in 300ml LB medium with ampicillin (100mg/L). IPTG was added to a final concentration of 0.4mM when the cells had grown to 2x10^8^ cells/ml (A600 = 0.5). The cells were then incubated at 37 °C for 4 hours and collected by centrifugation at 5000 × g for 10 minutes; the supernatant was decanted. The cells were resuspended in 100 ml column buffer consisting of 20 mM Tris-HCl, 200 mMNaCl, and 1 mM EDTA, followed by sonication in pulses of 15 seconds. The supernatant was harvested by centrifugation at 13000 × g for 20 minutes and then purified by using an amylase resin column. Depurated proteins were concentrated to at least 1mg/ml and cleaved by factor Xa to obviate the effect of MBP-tagexpressed by the vector on the enzyme activity analysis. The target protein was separated from MBP through anion exchange chromatography by using the pMAL™ protein fusion and purification system (NEB, New England) according to manufacturer’s instructions and then used for in vitro enzyme assay. Soluble fractions from the purification of pMAL-C5x-CtF3H were subjected to denaturing SDS-PAGE gel electrophoresis (10 % acrylamide) and visualized by Coomassie brilliant blue staining [46].

The *Ct*F3H protein was detected by the Modified Bradford Protein Assay Kit (Sangon biotech Co., Ltd) and quantifiedby the standard curve of BSA protein measured with the same method (Additional file 5: Figure S5).Then the *Ct*F3H activity assay was carried out at 30 °C for 10 minutes in a 100μl reaction containing 100 mMtricine (pH 7.5), 10 % (w/v) glycerol, 2 mg/ml ascorbic acid, 0.5 mg/ml catalase, 0.1mg/ml bovine serum albumin, 40μM FeSO4, 1mM 2-oxoglutaric acid, 100 μMnaringenin as substrate (all from Sigma-Aldrich, St. Louis, USA), and 50μg purified *Ct*F3H protein without MBP-tag [20, 47]. The reaction product was automatically sampled into a UPLC-Q-TOF/MS system equipped with an XBridge™BEH-C18 reverse phase column (2.1mm × 100mm, 2.5 μM). The solvents were (a) water with 0.1 % formic acid and (b) acetonitrile with 0.1 % formic acid. The separation procedure was as follows: 0–2 min, 5 % B; 2-15min, 95 %B. The product identification was based on a comparison of the chromatographic behavior and UV spectra with the use of authentic standards of naringenin and dihydrokaempferol under the same conditions. The kinetics of naringenin catalyzed by *Ct*F3H were determined by altering the naringenin concentration (10-800 μM) in the reaction buffer. Threereplicates were conducted for each concentration of naringenin. The kinetic parameters were obtained by applying the Michaelis–Menten equation with the use of the Graphpad Prism 5 software. A standard calibration curve was applied to calculate the quantity of the reaction product (Additional file 6: Figure S6).

### MeJA treatment

A 100μM solution of MeJA (Sigma-Aldrich) was sprayed onto healthy flowers of the ZHH0119 and XHH007 lines that opened on the first day when the corolla protruded from the sepals. In the control group, the flowers were sprayed with the same solution but without MeJA and then covered with plastic bags. To minimize the errors possibly resulting from the differences between individual plants, five flowers were sprayed for each concentration of the treatments and each flower were consecutively sprayed for five times. The treated flowers were enclosed by clear plastic bags to prevent the emission of volatile phytohormones and allow the elicitor solutions to be absorbed to a larger extent. After treatment for 0 h, 3 h, 6 h, and 12 h, the plastic bags were removed, and five samples of flowers at four time points were collected respectively, frozen immediately in liquid nitrogen and stored in freezers at −80 °C.

### Quantitative real-time PCR analysis of *Ct*F3H

Two groups of flowers were harvested after 0, 3, 6 and 12 hours for transcript analysis of CtF3H in the ZHH0119 and XHH007 lines. Total RNA of flowers were used to synthesize the first-strand cDNA for qRT-PCR (quantitative RT-PCR) analyses. PCR analysis was performed using specifc primers for *Ct*F3H gene (forward: ACACGAACCGACTATCCATA; reverse: GACCTATCTCCTTCATTCACTT) and *Ct*60s gene (forward:CATCCATTATCCAACAATC; reverse: AAGAGTAATCAGTCTCCA). qRT-PCR was done with the ABI7500 real-time PCR detection system (Applied Biosystems) using the TransStart Green qPCR supermix (TransGen Biotech, Shanghai, China) according to the manufacturer’s protocol. All qRT-PCR amplifcations were performed in three independent biological replicates and three technical replicates. The *Ct*60s gene (KJ634810) was selected as housekeeping gene (data not published). The relative expression level of *Ct*F3H was compared with its relative expression level of 0 h as reference using 2^-△△^Ct method.

### Metabolite extraction and UHPLC-Mass Spectrometry(MS)/Time of Flight(TOF) analysis

Five freeze-dried flowers precisely weighed out(4.0mg) were soaked in 1ml solvent (methanol: H_2_O = 6:4) overnight and then sonicated once for 40min. The extracts were centrifuged at 8000rpm for 10min; the supernatants were obtained and either stored at –80 °C or analyzed directly (4 μl) by using an ultra high-performance liquid chromatography(UHPLC) system (Agilent 1290 Infinity UHPLC; Agilent Technologies, Waldbronn, Germany) fitted with the Agilent 6538 UHD Accurate-Mass Q-TOF LC/MS (Agilent Technologies, Santa Clara, CA, USA) equipped with an ESI(electrospray ionization) interface. Chromatography separation was done with a Waters XSELECT™ HSS T3 C18 column (100 × 2.1mm, 2.5μm particle size) with a binary mobile phase consisting of (a) water with 0.1 % formic acid and (b) acetonitrile with 0.1 % formic acid. The column oven temperature was maintained at 40 °C. The ionization parameters included a gas temperature of 350 °C at a gas flow rate of 11 L/min; the voltages of the capillary, fragmentor, and skimmer1 were 4000V, 120V, and 60V, respectively, with a nebulizer at 45psi. The octopole RF peak voltage was set to 750V, and the reference masses were m/z 121.0509 and m/z 922.0098. The mass acquisition range was from 100-1100 amu, and the spectra were obtained in positive mode. The gradient elution of samples went from 95 % A to 5 % B in 2 min, 80 % A to 20 % B in 4 min, 79 % A to 21 % B in 6 min, 74 % A to 26 % B in 9 min, 60 % A to 40 % B in 11 min, 20 % A to 80 % B in 15 min, and 5 % A to 95 % B in 19 min, at a flow rate of 0.4ml/min. The compound identification was confirmed with the use of 12 authentic standards, namely: D-phenylalanine, hydroxysafflor yellow A, rutin, quercetin 3-β-D-glucoside, scutellarin, kaempferol-3-O-β-rutinoside, kaempferol-3-O-β-D-glucoside, dihydrokaempferol, luteolin, apigenin, carthamin, and kaempferol. Metabolite data were processed by using the MassHunter qualitative analysis software (Agilent) to obtain the peak fitting and abundance values. The metabolite accumulation in flowers after treatment with MeJA for 3 h, 6 h, and 12 h were respectively compared with that in flowers treated with MeJA for 0 h.

### Statistical analysis

Statistical analyses of metabolites accumulation in flower were performed with one way ANOVA. Each result was presented as the mean of at least three biological replicates ± standard deviation (SD) and the statistical signifcance was determined by *P*-value cut off of 0.05.

## Abbreviations

FAD, oleoylphosphatidylcholinedesaturase; F3H, flavanone 3-hydroxylase; PMT-39, pCAMBIA-1380-CaMV35S-MCS-EGFP-NOS; 2-ODD, 2–oxoglutarate-dependent dioxygenase; GFP, Green fluorescent protein; MBP, maltose binding protein; 2-OG, 2–oxoglutarate; MeJA, methyl jasmonate; LB, Luria-Bertani; MS, Murashige and Skoog; DAPI, 4’,6’-diamidino-2-phenylindole; UHPLC-MS/TOF, Ultra performance liquid chromatography- Mass Spectrometry/Time of Flight.

## Additional files

Additional file 1: Figure S1. Phylogenetic tree of amino acid sequences of F3Hs from different plant species. (JPG 50 kb) Additional file 2: Figure S2. SDS-PAGE analysis of recombinant *Ct*F3H protein without MBP- tag. (JPG 33 kb) Additional file 3: Figure S3. Kinetic analysis of purified CtF3H without MBP-tag. (JPG 32 kb) Additional file 4: Figure S4. The ECI profiles of flavonoids. (PDF 890 kb) Additional file 5: Figure S5. The standard curve of BSA protein measured by modified Bradford method. (JPG 36 kb) Additional file 6: Figure S6. The standard curve of dihydrokaempfreol and naringenin. (JPG 41 kb)
