# Supplementary figures and images for: Molecular characterization of flavanone 3-hydroxylase gene and flavonoid accumulation in two chemotyped safflower lines in response to methyl jasmonate stimulation
Source: BMC Plant Biol. 2016 Jun 10;16:132. doi: 10.1186/s12870-016-0813-5 (PMC4902928; doi:10.1186/s12870-016-0813-5)

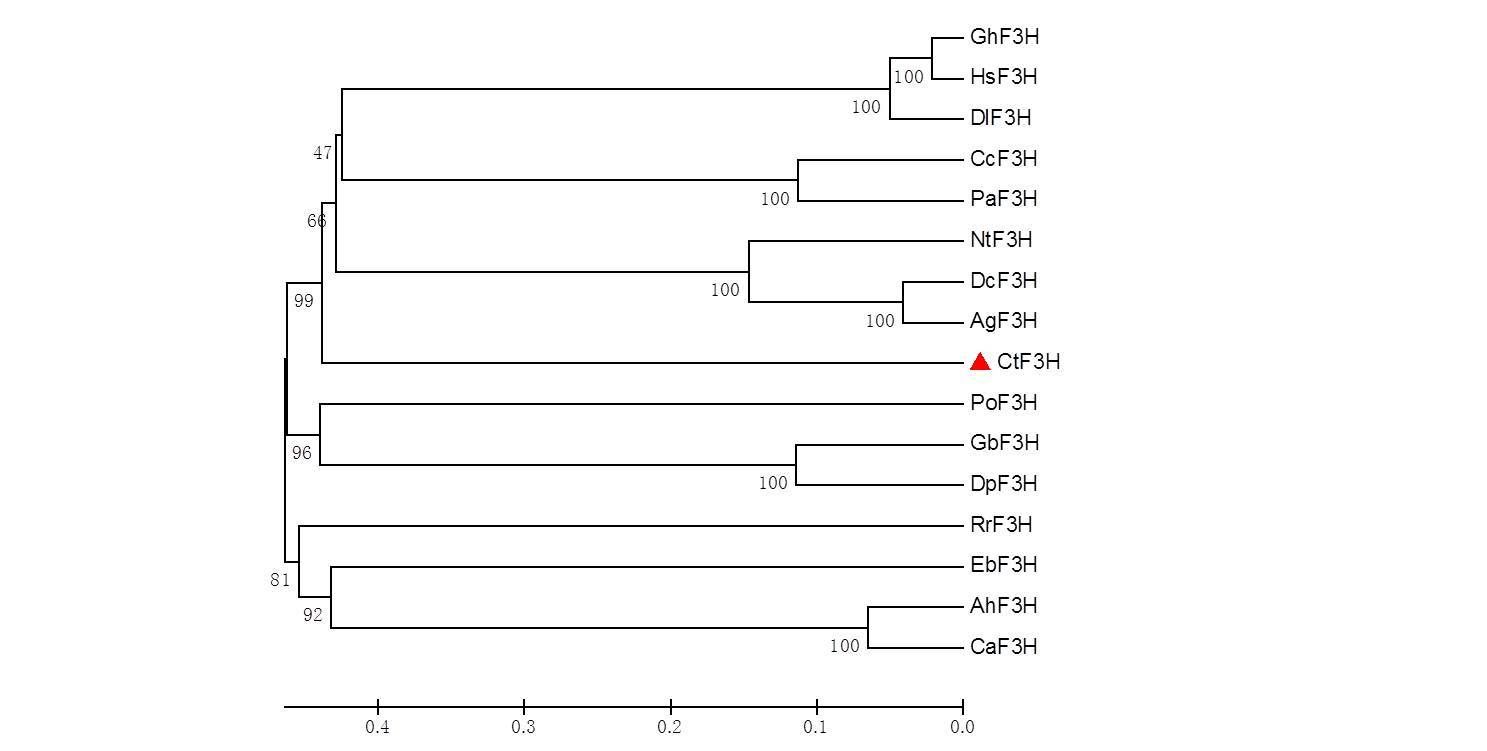

Supplement: Additional file 1: Figure S1. — Phylogenetic tree of amino acid sequences of F3Hs from different plant species. (JPG 50 kb) [file 12870_2016_813_MOESM1_ESM.jpg]

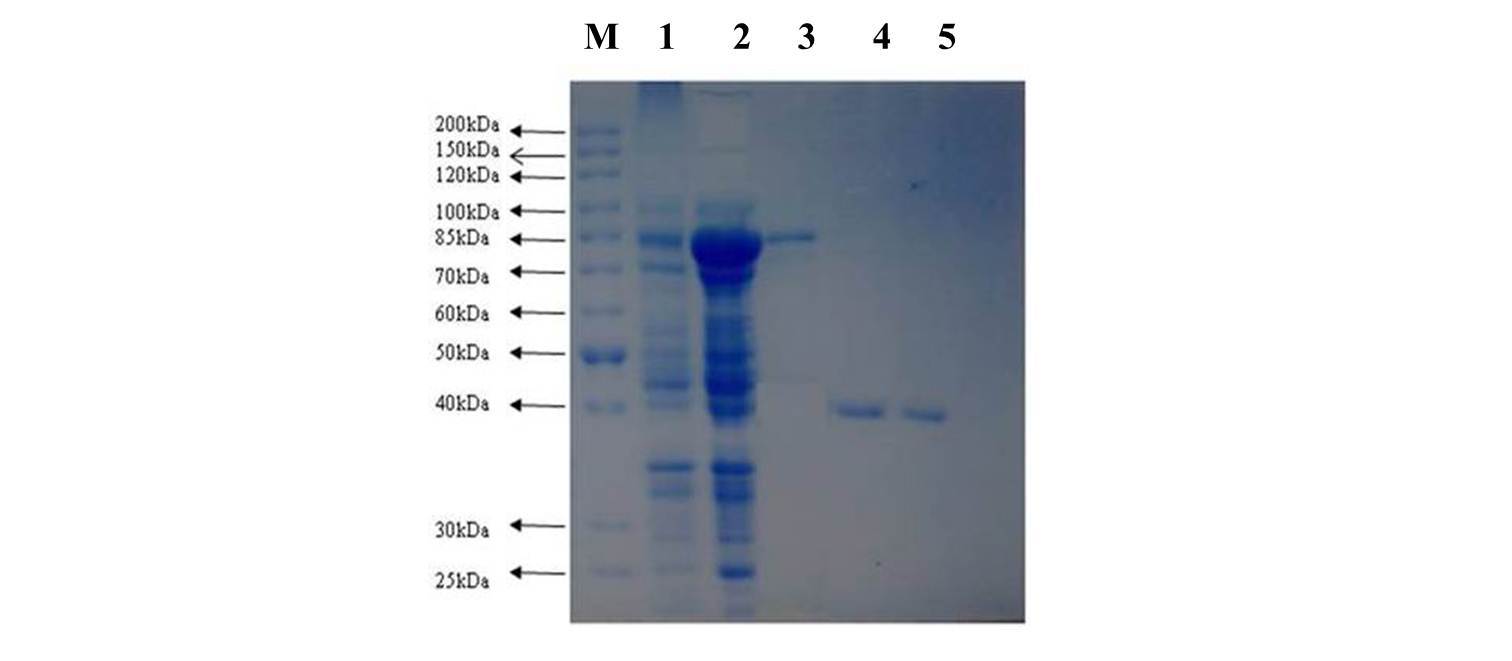

Supplement: Additional file 2: Figure S2. — SDS-PAGE analysis of recombinant CtF3H protein without MBP- tag. (JPG 33 kb) [file 12870_2016_813_MOESM2_ESM.jpg]

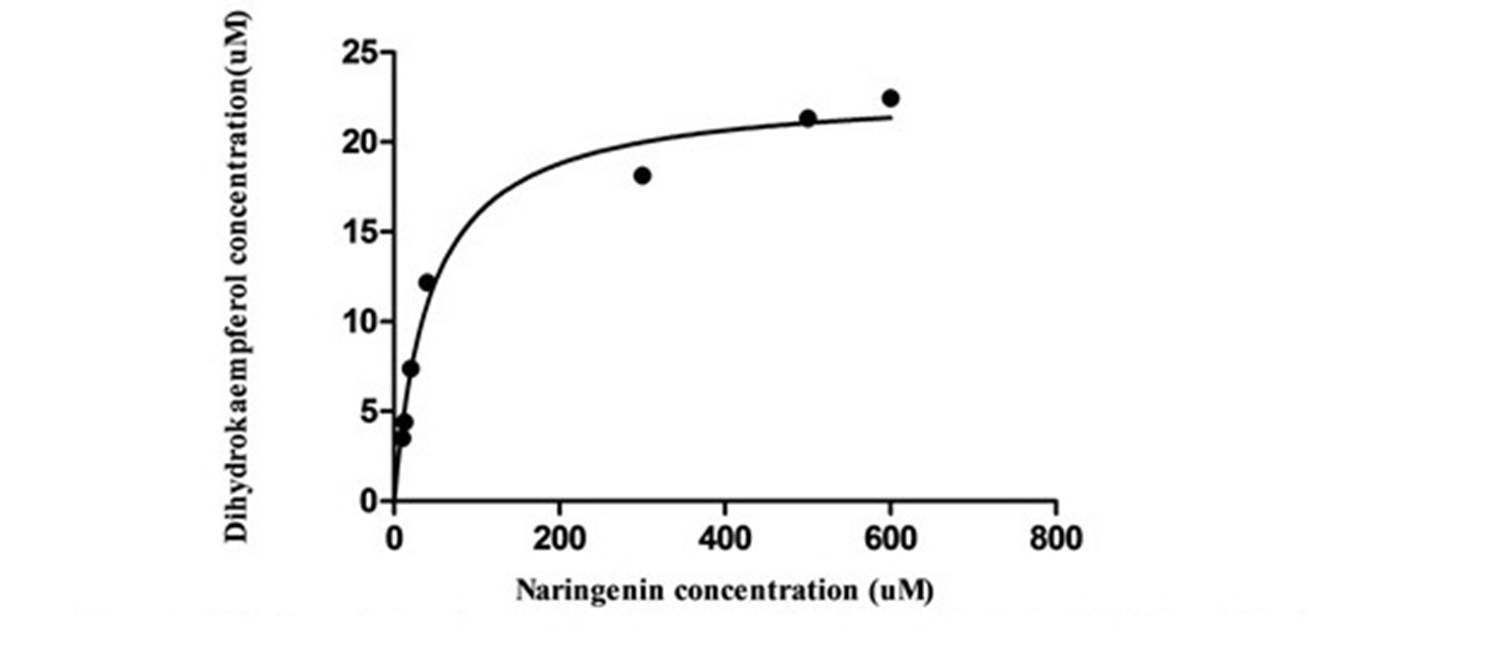

Supplement: Additional file 3: Figure S3. — Kinetic analysis of purified CtF3H without MBP-tag. (JPG 32 kb) [file 12870_2016_813_MOESM3_ESM.jpg]

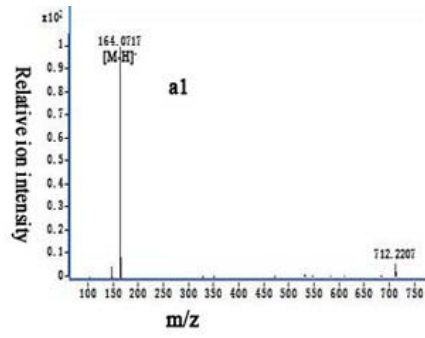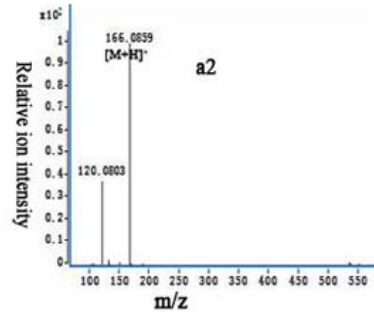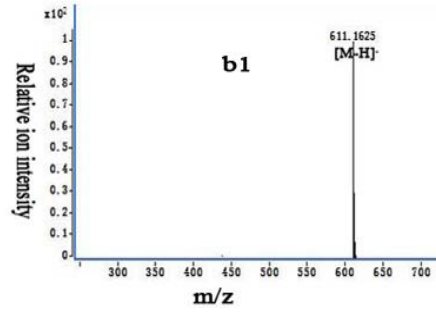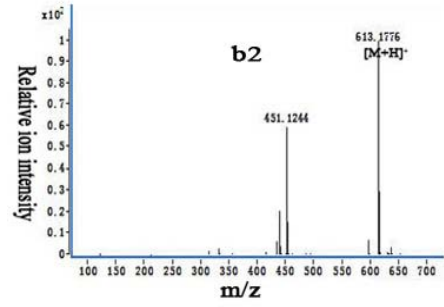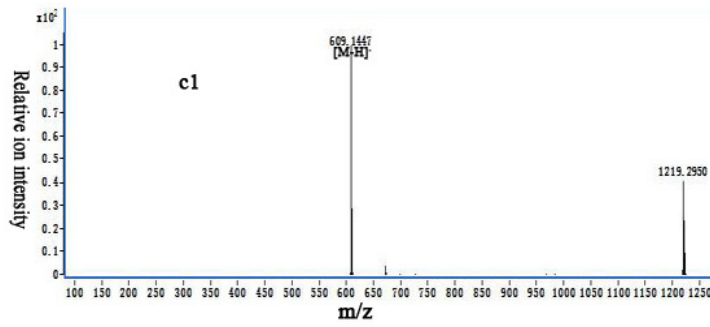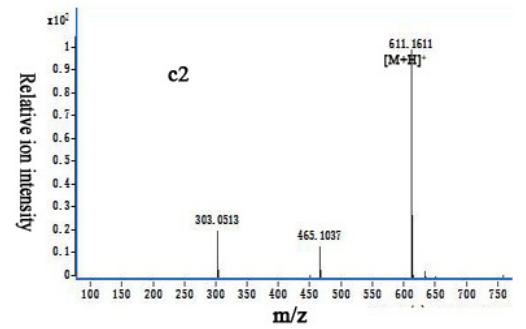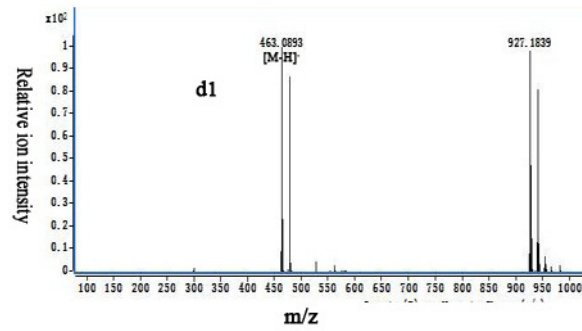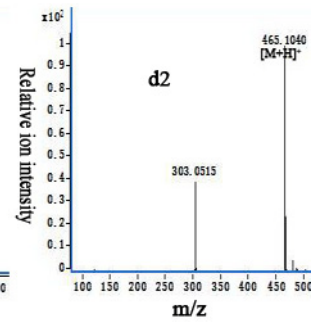

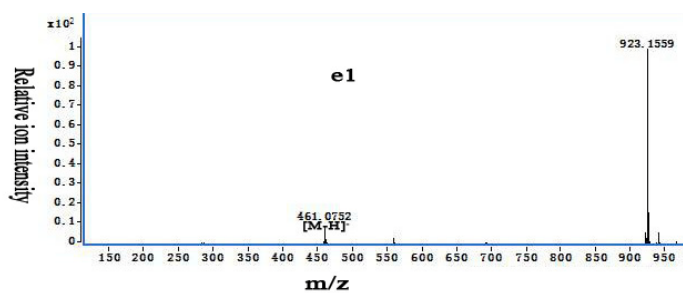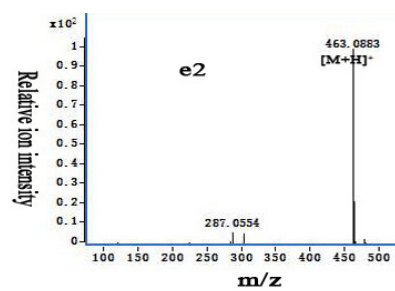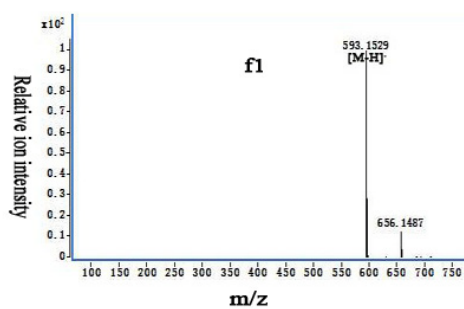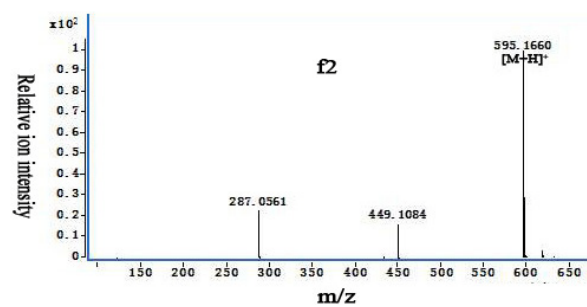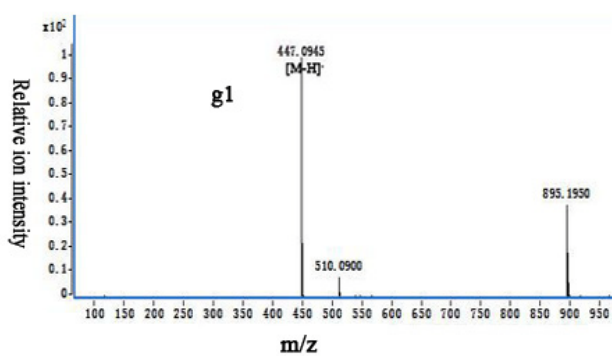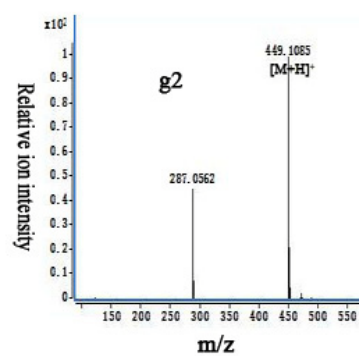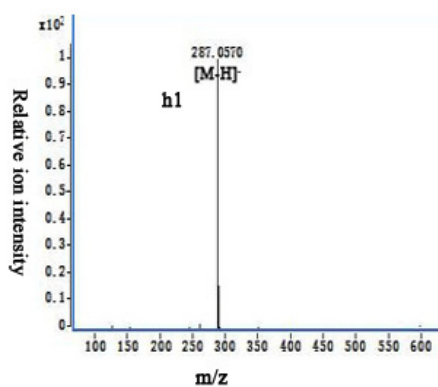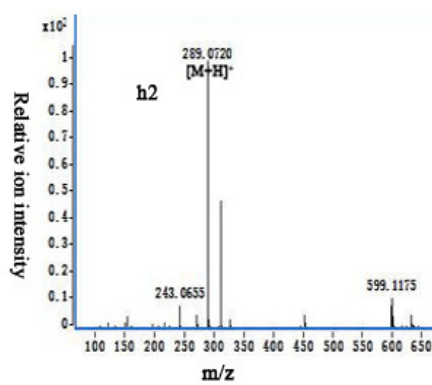

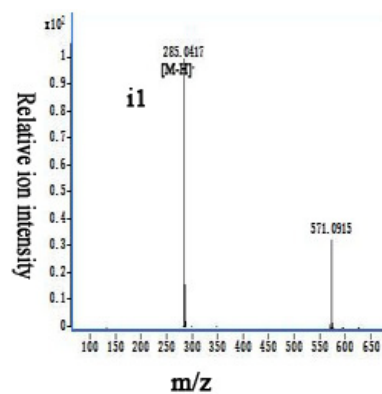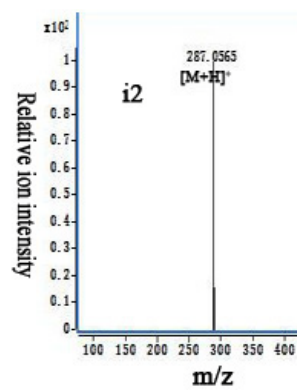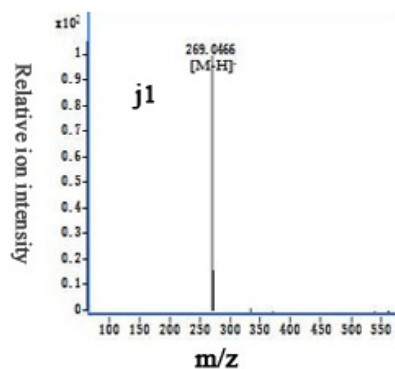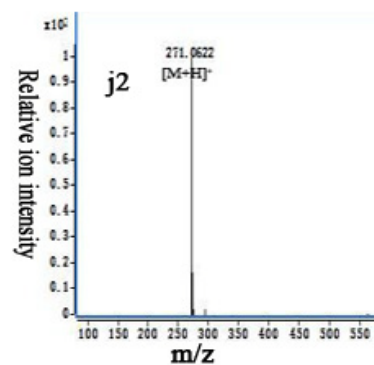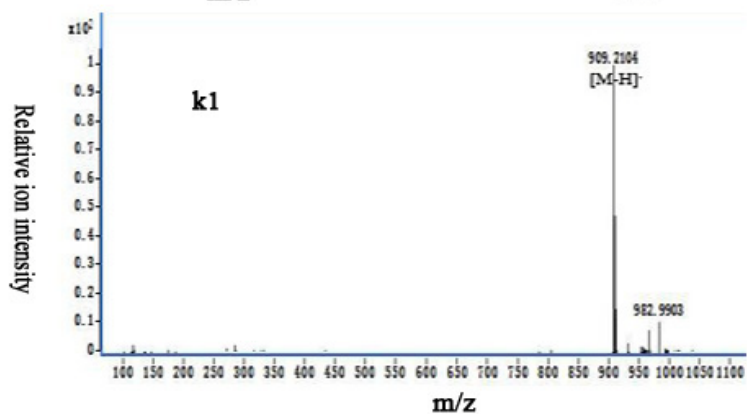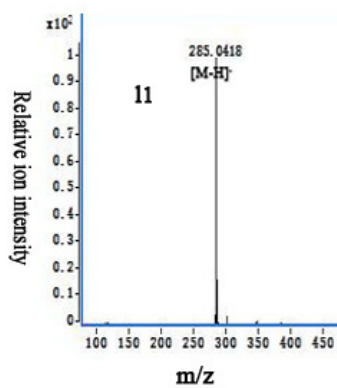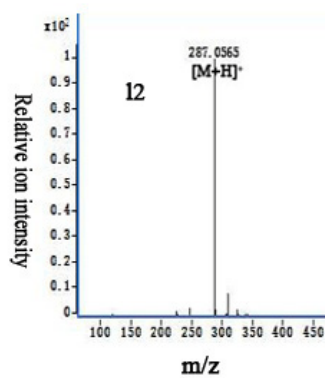

Supplement: Additional file 4: Figure S4. — The ECI profiles of flavonoids. (PDF 890 kb) [file 12870_2016_813_MOESM4_ESM.pdf]

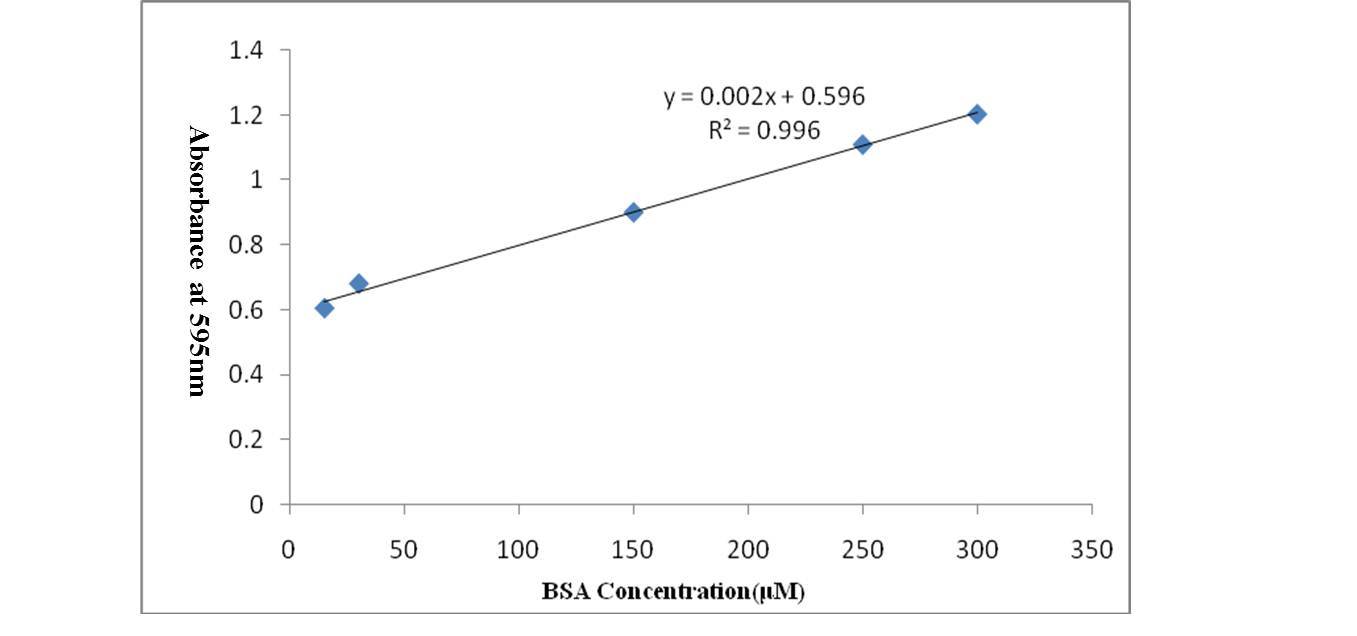

Supplement: Additional file 5: Figure S5. — The standard curve of BSA protein measured by modified Bradford method. (JPG 36 kb) [file 12870_2016_813_MOESM5_ESM.jpg]

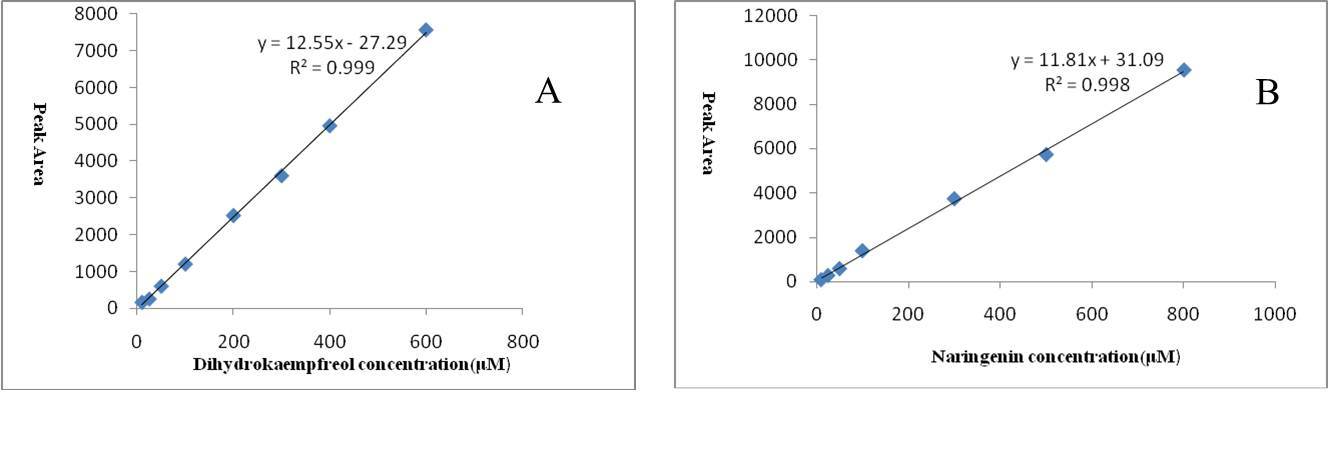

Supplement: Additional file 6: Figure S6. — The standard curve of dihydrokaempfreol and naringenin. (JPG 41 kb) [file 12870_2016_813_MOESM6_ESM.jpg]
